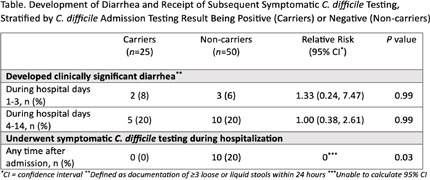# Impact of Clostridioides difficile Admission Screening in the Hematology-Oncology Unit on Infection Rates and Symptomatic Testing

**DOI:** 10.1017/ash.2025.197

**Published:** 2025-09-24

**Authors:** Christine Lucky, Lahari Thotapalli, Laura Rusie, Yoona Rhee, Michael Schoeny, Nicole Kraut, Alexandra Seguin, Brian Stein, Raul Rodriguez, Mary Hayden, Michael Lin

**Affiliations:** 1 Rush University Medical Center; 2 Medical College of Wisconsin

## Abstract

**Background:** Clostridioides difficile infection (CDI) disproportionately impacts hematology-oncology patients. In June 2022, our hospital implemented screening of asymptomatic patients admitted to the hematology-oncology unit to reduce CDI rates by early identification and isolation of C. difficile carriers. We evaluated the impact of admission screening on rates of CDI and compared incidence of diarrhea and subsequent symptomatic testing stratified by asymptomatic admission testing result. **Method:** During the intervention period (July 2022 – July 2024), asymptomatic patients admitted to the hematology-oncology unit were tested for C. difficile (perirectal swab, Cepheid GeneXpert®, Sunnyvale, CA). Guidelines for C. difficile symptomatic testing (unformed stool, Cepheid GeneXpert®) and treatment did not change between the baseline (May 2020 – May 2022) and intervention periods. Monthly CDI rates were calculated using CDC definitions based on clinical symptoms and positive C. difficile testing (community onset [CO] if positive in the first three hospital days, hospital-onset [HO] if day 4 or later). We performed an interrupted time-series analysis adjusted for repeated measures to compare CO-CDI and HO-CDI rates per 10,000 patient-days between baseline and intervention periods. The risk of developing diarrhea through hospital day 14 or being tested for symptomatic CDI during the intervention’s first year (July 2022 – June 2023) was analyzed using a cohort of asymptomatic C. difficile carriers and non-carriers in a 1:2 ratio, matched on hospital length of stay and date of admission. **Result:** The incidence rate ratio was 0.45 (P=0.10) for HO-CDI (Figure 1) and 0.15 (P=0.049) for CO-CDI (Figure 2) after screening implementation. During the first year of the intervention, 25 individuals were identified as asymptomatic C. difficile carriers by positive admission screen and were matched to a cohort of 50 asymptomatic non-carriers. There were no significant differences in development of diarrhea during hospital days 1-3 or days 4-14 between carriers and non-carriers (Table). None of the carriers received symptomatic C. difficile testing during hospitalization, compared to 20% of matched non-carriers (P=0.03). **Conclusion:** There was no significant change in HO-CDI rates and a statistically significant reduction in CO-CDI rates after implementation of C. difficile admission screening. Patients identified as carriers at time of admission were less likely to be tested for CDI during hospitalization than non-carriers, despite similar rates of diarrhea. Admission screening for C. difficile may reduce CDI rates through a variety of mechanisms; changes in provider testing behavior for patients previously screened for C. difficile may play a role.

**Figure:**
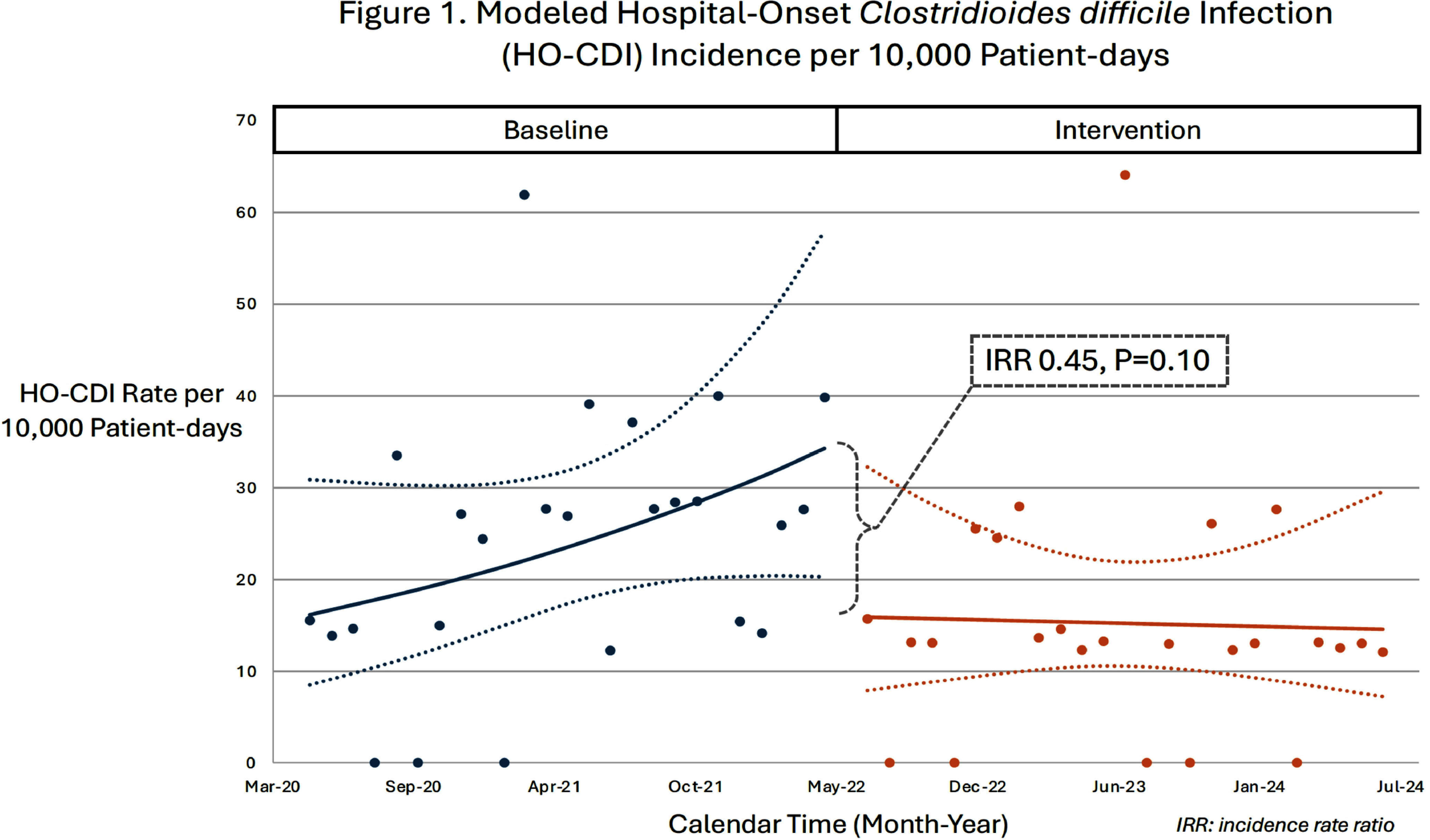

**Figure:**
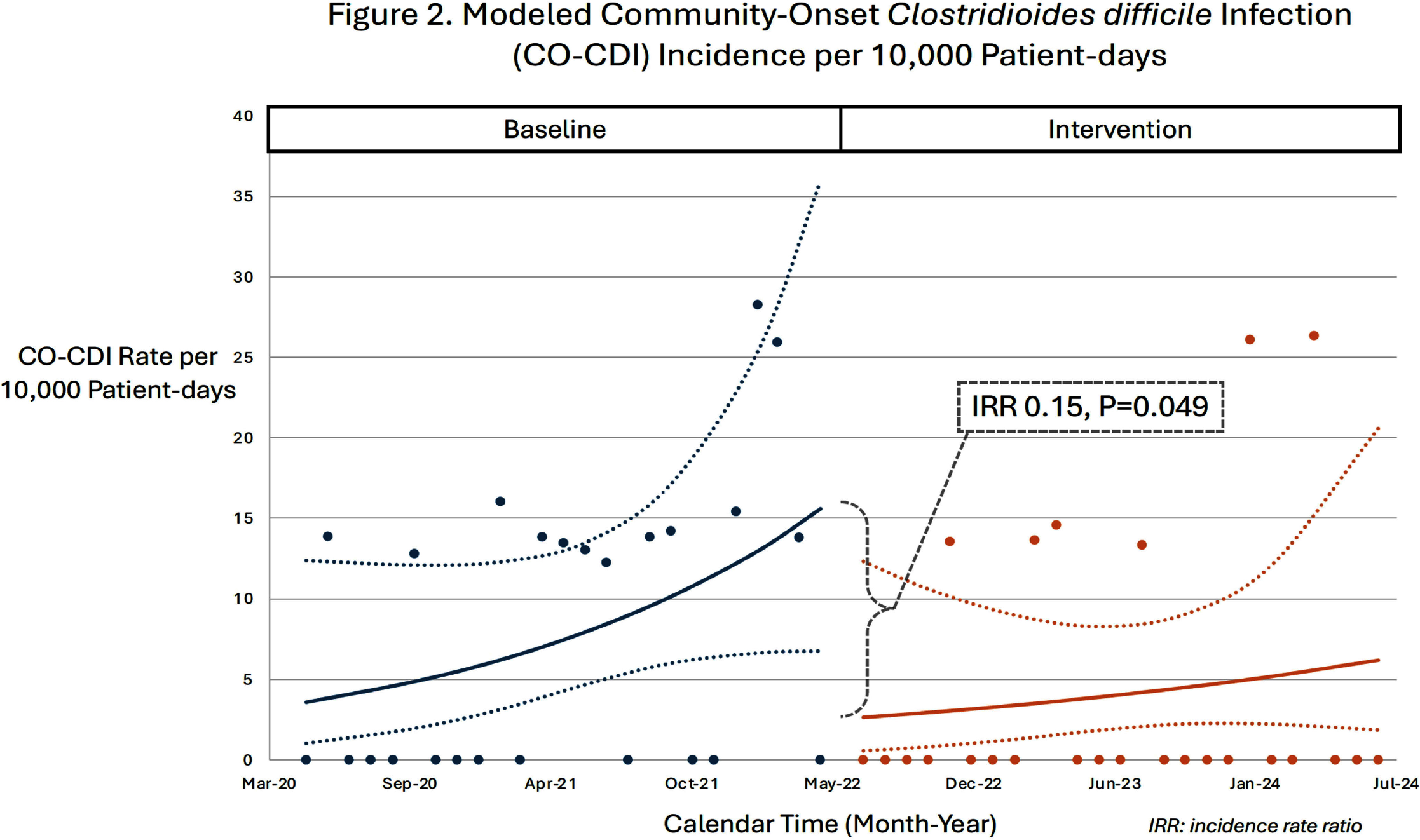

**Figure:**